# Preparation of ZnO Photocatalyst for the Efficient and Rapid Photocatalytic Degradation of Azo Dyes

**DOI:** 10.1186/s11671-017-1904-4

**Published:** 2017-02-21

**Authors:** Xiaoqing Chen, Zhansheng Wu, Dandan Liu, Zhenzhen Gao

**Affiliations:** School of Chemistry and Chemical Engineering/The Key Lab. for Green Processing of Chemical Engineering of Xinjiang Bingtuan, Shihezi University, Shihezi, 832003 People’s Republic of China

**Keywords:** ZnO, Azo dyes, Photocatalytic degradation, UV irradiation

## Abstract

Zinc oxide (ZnO) photocatalysts were synthesized by sol–gel method using zinc acetate as precursor for degradation of azo dyes under UV irradiation. The resultant samples were characterized by different techniques, such as XRD, SEM, and EDX. The influence of preparation conditions such as calcination temperature and composite ratio on the degradation of methyl orange (MO) was investigated. ZnO prepared with a composite ratio of 4:1 and calcination temperature of 400 °C exhibited 99.70% removal rate for MO. The effect of operation parameters on the degradation was also studied. Results showed that the removal rate of azo dyes increased with the increased dosage of catalyst and decreased initial concentration of azo dyes and the acidic condition is favorable for degradation. Furthermore, the kinetics and scavengers of the reactive species during the degradation were also investigated. It was found that the degradation of azo dyes fitted the first-order kinetics and superoxide ions were the main species. The proposed photocatalyst can efficiently and rapidly degrade azo dyes; thus, this economical and environment-friendly photocatalyst can be applied to the treatment of wastewater contaminated with synthetic dyes.

## Background

Synthetic organic dyes are used in the textile, paper, plastic leather, food, and other industries. About half of these dyes are azo compounds, such as methyl orange (MO), Congo red (CR), and direct black 38 (DB38), which contain chromophore (–N=N–) in their molecular structures [1]. However, effluents containing azo dyes are discharged into lakes, rivers, or ground waters during the dyeing process and contain many health hazards such as mutagenic and carcinogenic [2]. These dyes can lead to very serious environmental problems, due to their good stability under ambient conditions. Therefore, scholars have focused on eliminating azo dyes from wastewater to satisfy stringent environmental regulations. Up to now, various treatment methods such as physical methods and chemical methods have been investigated to remove azo dyes [3–7]. However, these methods cannot completely destroy contaminants and only transfer dyes from the solution to the adsorbent; as such, the dyes are transformed into their carcinogenic, mutagenic, or toxic intermediates, which cause secondary pollution. Thus, inexpensive and environment-friendly processes for the complete conversion of pollutants must be developed.

Recently, photocatalysis can be conveniently applied for their degradation of dye pollutants because it can mineralize organic dyes completely into H_2_O, CO_2_, and mineral acids without bringing secondary pollution. Metal semiconductor materials, such as TiO_2_ [8], ZnO [9], Fe_2_O_3_ [10], CdS [11], and ZnS [12], are used as photocatalyst. These cost-efficient, effective, and environment-friendly materials can be used to alleviate environmental problems. It is reported that among various semiconductors, zinc oxide (ZnO) exhibits higher efficiency in the photocatalytic degradation of some organic dyes than TiO_2_ [13, 14]. Therefore, it is extremely possible that ZnO will become another photocatalyst after TiO_2_, which is widely applied to treatment of contaminants.

ZnO is a representative n-type semiconductor, with a wide band gap of 3.37 eV and a high excitation binding energy of 60 meV [15], and produces electron–hole pairs under UV light or visible light irradiation. The electron and hole can interact with the O_2_ adsorbed on the surface of the photocatalyst and H_2_O to generate ·O_2_^−^ and ·OH, respectively, which can reduce and oxidize the organic contaminants completely into their respective end products (CO_2_ and H_2_O, respectively) [16, 17].

ZnO nanoparticles are synthesized through various techniques, such as hydrothermal synthesis [18], homogeneous precipitation [19], and sol–gel method [20]. Hydrothermal synthesis has many drawbacks, such as expensive equipment, large investment, large particle size, and poor dispersion [21]. However, the sol–gel method exhibits wide application potential not only due to simple operation and mild conditions but also because of the narrow size distribution and excellent crystalline structure of particles synthesized by sol–gel [22]. In recent years, research on ZnO has paid more attention on emphasizing the degradation of a separate azo dye over ZnO [1, 23, 24]. However, the degradation of ZnO for azo dyes containing different azo bonds has not been reported yet. Furthermore, some degradation conditions affecting the degradation of ZnO for different azo bonds dyes are worthy of discussion and analysis.

In this work, ZnO nanoparticles were prepared using the sol–gel method with zinc acetate as precursor for the degradation MO, CR, and DB38. The crystal structure and chemical properties of the samples were characterized using X-ray diffraction (XRD), scanning electron microscope (SEM), and energy-dispersive X-ray spectroscopy (EDX) analyses. Moreover, the photocatalytic activity of ZnO was evaluated using the degradation of azo dyes. The preparation conditions (calcination temperature and composite ratio) and degradation conditions (initial concentration of azo dye, dosage of ZnO, and initial pH) were also explored to analyze their effect on the degradation. The current study provides a basis for the application of ZnO as a photocatalyst to alleviate azo dye pollution.

## Methods

### Material

Zinc acetate was purchased from Tianjin Fuchen Chemical Reagent Co., Ltd. Oxalic acid was supplied by Tianjin Shengao Chemical Industry Limited Company. EtOH (anhydrous alcohol) was provided by Tianjin Fuyu Fine Chemical Co., Ltd. The selected properties of MO, CR, and DB38 are shown in Table 1. MO, CR, and DB38 were obtained from Tianjin Yong Sheng Fine Chemical Co., Ltd. All reagents were of analytical grade and used without further purification.

**Table 1:** Selected properties of azo dyes

Azo dyes	Structure	Formula	Molecular weight (g/mol)	Dye type	MW/S	Azo bond
MO		C_14_H_14_N_3_SO_3_Na	327.33	Anionic	163.67	1
CR		C_32_H_22_N_6_Na_2_O_6_S_2_	696.68	Anionic	348.34	2
DB38		C_34_H_25_N_9_Na_2_O_7_S_2_	781.73	Anionic	390.87	3

MW/S is the ratio of the molecular weight of dyes to the number of sulfonic groups in the dye

### Preparation of ZnO

ZnO was synthesized by the conventional sol–gel method. In a typical experiment, 2.196 g (0.01 mol) of zinc acetate was dissolved in 60 mL of EtOH and stirred at 60 °C for 30 min to obtain solution A. Solution B was prepared by dissolving 2.520 g (0.02 mol) of oxalic acid dehydrate in 80 mL of EtOH and stirred at 50 °C for 30 min. Solution B was added to the warm solution A dropwise and continuously stirred for 1 h. A white sol was obtained and aged to form a gel, which was dried at 80 °C for 24 h. Finally, ZnO was obtained by thermal treatment at different calcination temperatures of 300, 400, 500, and 600 °C. Solutions with different composite ratios (molar ratio of oxalic acid to zinc acetate), ranging from 2 to 5, were prepared while keeping the ratio of zinc acetate at 0.01 mol.

### Characterizing Methods

XRD patterns of all photocatalysts were collected in the region 2*θ* = 10°–80° using a Rigaku GiegerFlex D/Max B diffractometer with Cu–K*α* radiation. The surface morphology of the samples was examined using SEM (JSM-6490LV, Japan) analysis at accelerating voltages of 20 kV. Elemental analysis of the sample was carried out using energy-dispersive X-ray spectroscope (EDX) (EDAX, GENESIS).

### Photocatalytic Activity

MO, CR, and DB38 were initially dissolved in water to prepare the 200 mg/L stock solution. The concentrations of various degradation solutions were measured by a UV–vis spectrophotometer (UV-5100). The concentrations of MO, CR, and DB38 were calculated based on the following calibration equations, respectively: (1) at 466 nm, (2) at 500 nm, and (3) at 595 nm. *C* = 0.0350A_466_ (1), and *R* ^2^ was equal to 0.9993. *C* = 0.0252A_500_ (2), and *R* ^2^ was equal to 0.9994. *C* = 0.0048A_595_ (3), and *R* ^2^ was equal to 0.9990.

The photocatalytic activity of ZnO was evaluated with a photoreaction system using the degradation of MO under UV irradiation at room temperature using a 1000-W UV lamp with 365-nm wavelength. In a typical process, 10 mg of photocatalyst was added to 50 mL of aqueous solution containing dye with a concentration of 30 mg/L. Then, the solution was kept in the dark for 30 min to reach adsorption–desorption equilibrium of the dye on the ZnO surface before irradiation. Next, the suspension was exposed to UV lamp to degrade the dye. The distance between the reactor and lamp is 8.5 cm. During the reaction, the reaction solution was stirred continuously. Each sample was taken out at a given time interval and immediately centrifuged at 10,000 rpm for 15 min to remove photocatalyst particles for analysis. Finally, the absorbance of the dye in the supernatant liquid was recorded by a UV-5100 spectrophotometer at the maximum absorption wavelength of the dye. The removal rate (*η*) of the dye can be calculated as follows:

$$ \eta =\frac{C_0-{C}_t}{C_0}\times 100\% $$

where *C*_0_ and *C*_t_ are the concentrations of the dye after self-photolysis and different irradiation times, respectively.

### Experiment of Radical Scavenger

To further study the photocatalytic mechanism of photocatalyst, main reactive species (radicals and holes) were detected through radical scavenging experiments in the photocatalytic process. The holes (h^+^), hydroxyl radical (·OH), and superoxide radical (·O_2_^−^) are trapped by adding ammonium oxalate (AO) (h^+^ scavenger), tert-butanol (*t*-BuOH) (·OH scavenger), and *p*-benzoquinone (*p*-BQ) (·O_2_^−^ scavenger) into the reaction solution, respectively, during the process of photocatalytic degradation. Typically, 10 mg of ZnO and 10 mM of radical scavengers were placed into 50 mL of 30 mg/L dye solution; then, the suspension was irradiated using the UV lamp for the same time. Finally, the removal rate (*η*) of the dye can be calculated to determine the main role of active species.

## Results and Discussion

### Effect of Preparation Conditions of ZnO on MO Degradation

#### Calcination Temperature

**Fig. 1:**
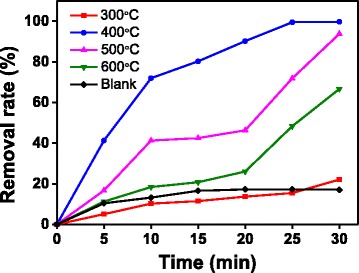
Effect of calcination temperature on the properties of ZnO (30 mg/L initial concentration of MO solution, 0.2 g/L ZnO, and pH = 6.8)

Figure 1 shows the comparison of the activities of the photocatalysts prepared at different calcination temperatures (300, 400, 500, 600 °C). The blank test of self-degradation of MO was also conducted. The removal rate of MO was low in the blank condition, and the highest removal rate was 17.22%. The removal rate of MO over ZnO initially increased, then decreased with the increase of the calcination temperature. At the calcination temperature of 400 °C, the removal rate reached 99.70%, which is higher than that of the other prepared ZnO samples. The degradation efficiency of the ZnO samples for MO followed the order 400 °C > 500 °C > 600 °C > 300 °C after UV irradiation for 30 min, which may be related to the particle size of the photocatalyst.

#### Composite Ratio

**Fig. 2:**
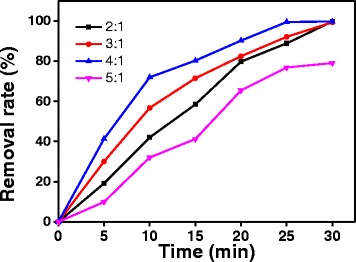
Effect of the complexation ratio on the properties of ZnO prepared at 400 °C (30 mg/L initial concentration of MO solution, 0.2 g/L ZnO, and pH = 6.8)

ZnO was prepared at different composite ratios (2:1, 3:1, 4:1, 5:1) to determine their influence on photocatalytic degradation of MO. The experimental results are shown in Fig. 2. The results indicate that the removal rate of MO over ZnO has reached 99.73, 99.29, and 99.70%, respectively, when the composite ratio was increased from 2:1 to 4:1 and after the reaction time of 30 min. When the composite ratio was further increased to 5:1, the removal rate decreased. However, at UV irradiation time of 25 min, the removal rate of MO on ZnO with composite ratio of 4:1 has reached 99.45%. Therefore, an appropriate composite ratio might improve the crystallinity and size of the particles; hence, the removal rate of MO was increased.

### Characterization

#### XRD Analysis

**Fig. 3:**
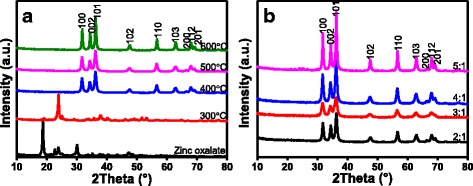
XRD patterns of ZnO prepared at different calcination temperatures (**a**) and different complexation ratios (**b**)

Figure 3 shows the XRD patterns used to characterize the crystal phases and crystallinity of the photocatalysts. Figure 3a illustrates the XRD patterns of ZnO prepared with zinc oxalate as precursor at different calcination temperatures by sol–gel process. The crystalline reflection of the samples corresponds to zinc oxalate and the diffraction data agreed well with the standard card JCPDS 037-0718 [20]. The precursor was not able to decompose into ZnO after calcination at 300 °C, because precursor decomposition could be occurred from 360 to 420 °C [20]. The characteristic diffraction peaks of the samples indicate the hexagonal wurtzite structure of ZnO after calcination at 400, 500, and 600 °C. It was clear that characteristic crystalline reflections of ZnO at 31.81°, 34.44°, 36.31°, 47.602°, 56.62°, 63.01°, 66.48°, 67.97°, and 69.19° correspond to the (100), (002), (101), (102), (110), (103), (200), (112), and (201) planes, respectively. The crystalline reflection data show good agreement with the standard card JCPDS for ZnO (JCPDS 36-1451) [3]. Besides, no reflections of other phases were detected, which indicated the high purity of the prepared sample.

All XRD crystalline reflections of ZnO prepared at various composite ratios also showed that the hexagonal wurtzite structure of ZnO did not change (Fig. 3b). The crystalline sizes of the samples were calculated by Scherrer equation, and the data were listed in Table 2.

**Table 2:** Crystallite size of ZnO nanoparticles under different conditions

Sample no.	Composite ratio	Calcination temperature (°C)	Crystallite size (nm)
1	2:1	400	23.60
2	3:1	400	25.28
3	4:1	400	22.56
4	5:1	400	35.00
5	4:1	300	—
6	4:1	500	33.29
7	4:1	600	50.10

— indicates that ZnO nanoparticles were not be formed and crystallite size could not be calculated

$$ d=\frac{k\lambda}{\beta \cos \theta} $$

where *d*, *k*, *λ*, *β*, and *θ* are the crystal size, Scherer constant (0.89), X-ray wavelength (0.154 nm), the peak full width at half maximum (FWHM), and the Bragg diffraction angle corresponding to ZnO (101) reflection at 36.31°, respectively. The crystal size of ZnO nanoparticles prepared with the composite ratio of 4:1 and calcination temperature of 400 °C was 22.56 nm, which was the smallest among all the samples. As the calcination temperature increased from 400 to 600 °C, the aggregation of the ZnO particles was enhanced and the average particle size increased from 22.56 nm to 50.51 nm. These results were in good agreement with performance experiments of ZnO prepared at different calcination temperatures. The decrease of the removal rate of MO over ZnO nanoparticles prepared at high calcination temperature could be the formation of a larger particle [25]. The possible reason is that ZnO with smaller particle size has more active sites when the amount of photocatalyst is the same, thereby promoting the formation of radicals and the adsorption of azo dyes on the photocatalyst surface. Therefore, ZnO prepared at 400 °C exhibits the smallest particle size and the highest photocatalytic activity among all the samples tested.

#### SEM

**Fig. 4:**
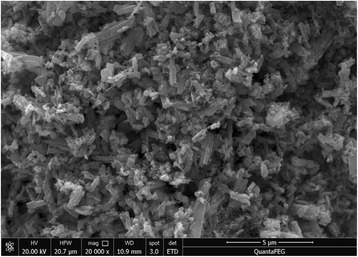
SEM image of ZnO prepared with a complexation ratio of 4:1 and calcination temperature of 400 °C

The further morphologic and structural characterization of the prepared ZnO particles was investigated through SEM analysis. The SEM image of the ZnO particles is shown in Fig. 4. The results indicate that synthesized ZnO by sol–gel method has a rod-like shape, which is typical morphology of ZnO particles. It is similar to the report of Chen et al. [26].

#### EDX

**Fig. 5:**
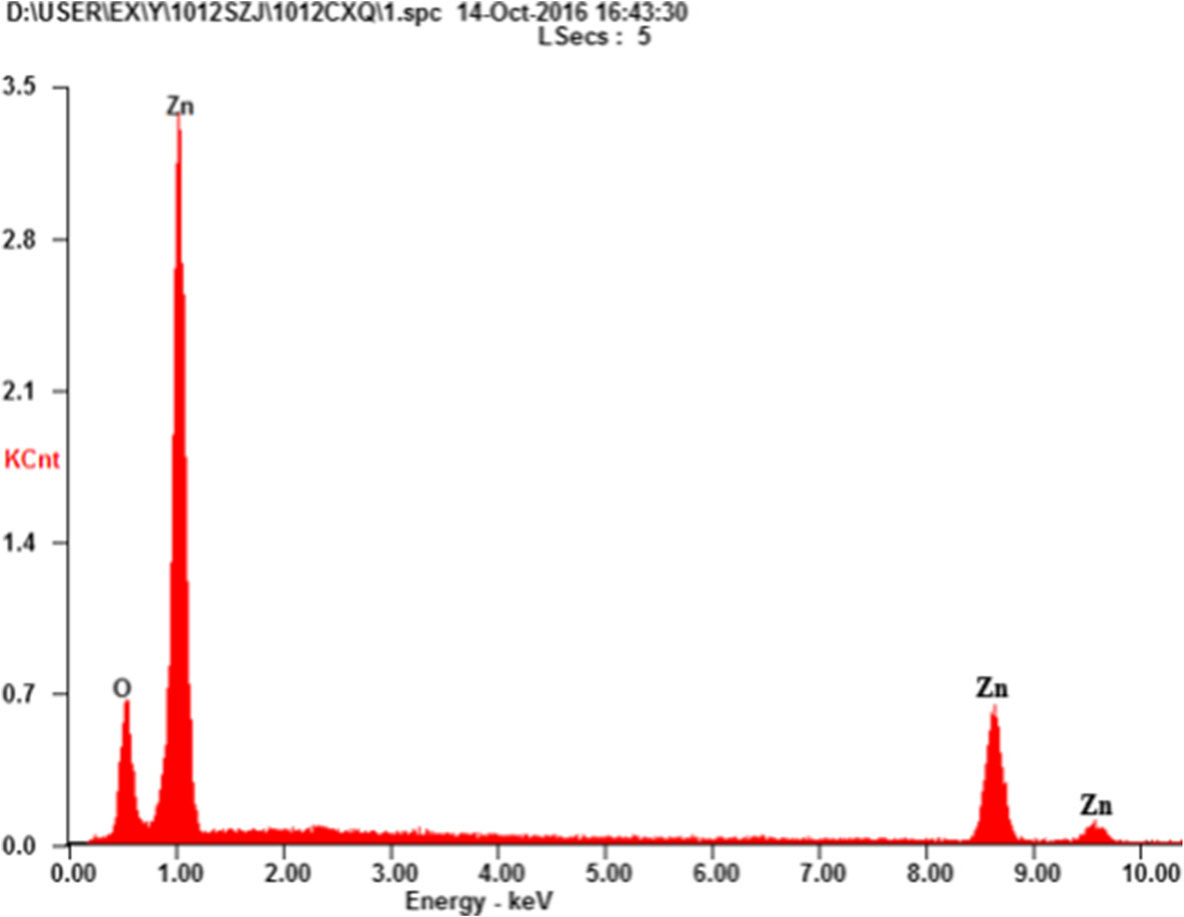
EDX spectra of ZnO prepared with the complexation ratio of 4:1 and calcination temperature of 400 °C

In order to further confirm that prepared products are pure ZnO without any impurity, EDX analysis was examined as presented in Fig. 5. Peaks assigned to Zn and O were found, but no impurity peaks were detected, which further confirmed that the synthesized ZnO is pure and consists of only Zn and O. The weight and atomic percentage of Zn and O are presented in Table 3. The weight percentages of Zn and O are 81.97 and 18.03%, respectively, which indicated that synthesized photocatalyst is only composed of Zn and O without other elements. The atomic percentages of Zn and O were near the approximate stoichiometric ratio of 1:1. Similar results have been reported [3].

**Table 3:** Weight% and atomic% results of ZnO under the optimal conditions

Element	Wt%	At%
O K	18.03	47.33
Zn L	81.97	52.67

### Effect of Operating Parameters on the Photodegradation of Azo Dyes

#### Initial Concentration

**Fig. 6:**
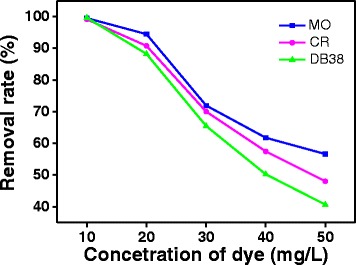
Effect of initial concentration on the photodegradation activities of ZnO prepared with the complexation ratio of 4:1 and calcination temperature of 400 °C (10 min of irradiation time, 0.2 g/L ZnO, and pH = 6.8)

To study the effect of azo dye initial concentrations on the photodegradation activity, the initial concentrations of MO, CR, and DB38 were changed from 10 to 50 mg/L. Results on the comparison of removal rates of the dyes after 10 min of reaction time are shown in Fig. 6. When other conditions were kept constant, with the increase of initial concentration, the removal rates of MO, CR, and DB38 decreased significantly from 99.53, 99.14, and 99.65% to 56.61, 48.03, and 40.64%, respectively. Therefore, the removal efficiency of dye could be enhanced by the lower initial concentration of the dye. This is parallel to the result in Thomas et al.’s study [23]. This may be explained that more and more dye molecules were adsorbed on the surface of the photocatalyst, when initial concentration of the dye was increased. Because many active sites were occupied by the dye molecules, the adsorption of O_2_ and OH^−^ on the photocatalyst was decreased, which leads to reduced generation of radicals. Furthermore, the photons were blocked before reaching the photocatalyst surface; hence, the adsorption of photons was decreased by the photocatalyst. Accordingly, the removal rate reduced at high initial dye concentrations [3, 27].

#### Dosage of ZnO

**Fig. 7:**
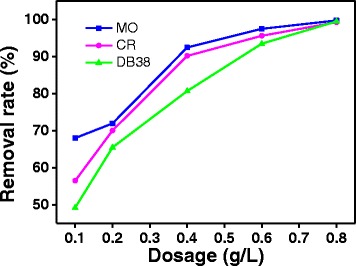
Effect of dosage on the properties of ZnO prepared with the complexation ratio of 4:1 and calcination temperature of 400 °C (10 min irradiation time, 30 mg/L initial concentration of solution, and pH = 6.8)

Figure 7 shows the removal rate of the azo dye (30 mg/L) in the presence of a photocatalyst of different concentrations (0.1–0.8 g/L). The photodegradation efficiency of MO, CR, and DB38 increased from 68.00, 56.49, and 49.25% to 99.70, 99.21, and 99.45%, respectively, when the photocatalyst dosage was varied from 0.1 to 0.8 g/L. The results are similar to the study of Mondal et al. [28]. When the photocatalyst amount was increased, more and more active sites were found on the photocatalyst surface, which leads to the increase of formation of radicals. Therefore, the high dosage could improve the degradation efficiency of the azo dye [29, 30].

#### Initial pH of Solution

**Fig. 8:**
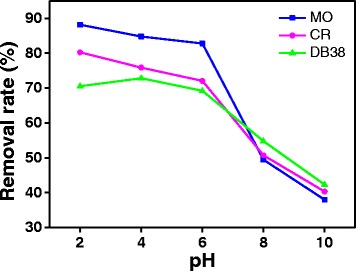
Effect of pH on the properties of ZnO prepared with the complexation ratio of 4:1 and calcination temperature of 400 °C (10 min irradiation time, 30 mg/L initial concentration of solution, and 0.2 g/L ZnO)

Usually, wastewater from industries exhibits a wide pH range. Meanwhile, the pH of the dye aqueous solution is a significant factor in the photodegradation processes. Figure 8 shows the removal rates of azo dyes on ZnO at pH levels of 2.0, 4.0, 6.0, 8.0, and 10.0, which were adjusted using HCl and NaOH. The removal ratio is high when the initial pH of the dye solution is acidic. By contrast, the removal ratio of dyes is very low at high pH levels. It is similar to the reports of Zhu et al., Thomas et al., and Paz Diego et al. [1, 23, 24]. This phenomenon could be attributed to the properties of dyes and the surface-charge properties of photocatalysts which were related to zero charge. The surface of ZnO became positively charged at a pH lower than pH_zpc_; however, a negative charge is expected when the pH is higher than pH_zpc_. At acidic pH, a large number of O_2_ was reduced into ·O_2_^−^ radicals by photoelectrons since the positively charged surface of the photocatalyst is conducive to the transfer of photoelectrons to the photocatalyst surface. Table 1 confirms that MO, CR, and DB38 are anionic dyes. At low pH values, the high removal ratio of dyes was obtained due to electrostatic attraction between dye anions and the photocatalyst surface with positive charge, resulting in the increase of degree of adsorption and photodegradation. On the contrary, at high pH, electrostatic repulsion may occur between the dye anions and the negatively charged photocatalyst surface, resulting in negligible adsorption. A similar result has been reported by Bagheri et al. [27]. In acidic condition, MO-, CR-, and DB38-containing sulfonic groups can be easily ionized to form anionic dyes, thereby enhancing the adsorption of dye anions on the ZnO surface. Moreover, in acidic condition, the molecular structure of azo dyes might change into the quinoid structure, which is unstable and could be easily broken. While, in alkaline condition, the dye molecule exists in azo formula, which is firm and difficult to be decomposed [1]. Therefore, it is more favorable to degrade dyes over ZnO in acidic solution.

### Degradation Kinetics

**Fig. 9:**
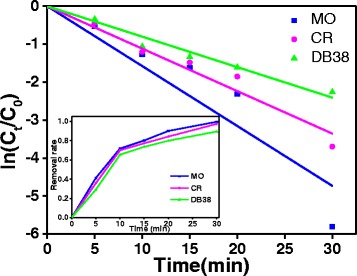
Photocatalytic degradation kinetic curves for photocatalytic degradation of MO, CR, and DB38 over ZnO under UV irradiation (30 mg/L initial concentration of solution, 0.2 g/L ZnO, and pH = 6.8)

The removal rates of various azo dyes on ZnO (0.2 g/L) were estimated under UV irradiation (Fig. 9 inset). For comparison, the degradation rates of MO, CR, and DB38 were observed under UV irradiation for 30 min in the absence of photocatalysts and reached 99.70, 97.53, and 89.59%, respectively, indicating that the photocatalytic degradation of monoazo dye is the highest under UV irradiation [31]. Usually, photocatalytic processes are carried out only in water because the radicals can only react with the azo dyes dissociated. Therefore, the degradation of dyes is likely related to their dissociation degree of azo dyes in water. Firstly, the association property of azo dyes was appreciably strengthened with the increase of MW/S, resulting in decreased dissociation degree. Hence, azo dyes are hardly degraded in water. Secondly, the distribution of the dyes in water increased with the increased molecular weight of azo dyes, likely caused by the increased molecular weight of azo dyes as increased azo bonds, which leads to decreased degradation rate of azo dyes. Therefore, the degradation rate of azo dyes decreased with the increase of the azo bond and MW/S. The azo bond and MW/S of MO, CR, and DB38 are listed in Table 1.

The kinetics of dye degradation was estimated. The kinetics models of the pseudo-first-order model were tested to determine the kinetics rate in the degradation process of MO, CR, and DB38 onto the ZnO nanoparticles and are commonly expressed as the flowing equation:

$$ \ln \left(\frac{C_t}{C_0}\right)= k t $$

where *k* is the photodegradation rate constant (min^−1^) and *C*_*0*_ and *C*_*t*_ are the concentrations (mg/L) of dye after self-photolysis and at different irradiation times, respectively. The linear fit between ln(*C*_*t*_/*C*_0_) and reaction time *t* of different dyes follows a pseudo-first-order kinetics behavior [13, 30]. All results are presented in Fig. 9. All correlation coefficients (*R* ^*2*^) were higher than 0.9293 (Table 4), which indicates that the photodegradation of dyes fits well with the kinetic model. The rate constants of MO, CR, and DB38 are 0.1578, 0.1119, and 0.0803 min^−1^, respectively.

**Table 4:** First-order kinetic constants and relative coefficients for photocatalytic degradation of azo dye over the photocatalysts

Azo dye	*k* (min^−1^)	*R* ^*2*^
MO	0.1578	0.9293
CR	0.1119	0.9814
DB38	0.0803	0.9877

### Stability of Photocatalyst ZnO

**Fig. 10:**
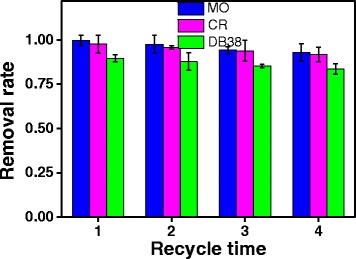
Recycled photoactivity testing of ZnO for degradation of MO, CR, and DB38 under UV irradiation (30 min irradiation time, 30 mg/L initial concentration of solution, 0.2 g/L ZnO, and pH = 6.8)

In addition to photocatalytic property, the stability of photocatalysts is important in large-scale processes. Hence, to investigate the stability of ZnO photocatalysts, recycling experiments of ZnO for photocatalytic degradation of MO, CR, and DB38 under UV irradiation were carried out and the results are listed in Fig. 10. The photocatalyst was collected after each cycle by centrifugation, then washed with distilled water and ethanol and dried in an oven at 80 °C. The sample was then reused for subsequent degradation. As can be seen, the removal rate of MO, CR, and DB38 decreased from 99.70, 97.53, and 89.59% to 92.88, 91.69, and 83.48%, respectively, after four cycles. The photocatalytic activity of ZnO only minimally decreases, due to the unavoidable loss of photocatalysts during the cycle processes. Therefore, the ZnO photocatalyst remains a high photocatalytic activity and stability under UV irradiation for a long time.

### Mechanism of Photodegradation

**Fig. 11:**
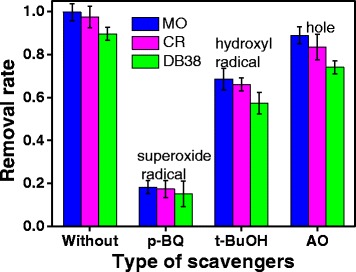
Removal ratio of MO, CR, and DB38 over ZnO in the presence of various scavengers (30 min irradiation time, 30 mg/L initial concentration of solution, 0.2 g/L ZnO, and pH = 6.8)

The photocatalytic reaction generally includes photoexcitation, charge separation and migration, and surface oxidation–reduction reactions [32]. The reactive species generated during illumination of photocatalysts are h^+^, OH^−^, and ·O_2_^−^. To understand the mechanism of ZnO for degradation dyes, it is necessary to detect which reactive species plays a major role in the photocatalytic degradation process. During the photodegradation of dyes over ZnO, the h^+^, ·OH, and ·O_2_^−^ are eliminated by adding AO (h^+^ scavenger) [33], *t*-BuOH (·OH scavenger) [34], and *p*-BQ (·O_2_^−^ scavenger) [35] into the reaction solution, respectively. Figure 11 shows the degradation rate in the presence and absence of the scavengers. The addition of *t*-BuOH and AO only slightly changed in the photocatalytic degradation of azo dyes. However, the removal rates of MO, CR, and DB38 are considerably reduced to 18.31, 17.48, and 15.21% with the addition of a scavenger for ·O_2_^−^ (*p*-BQ). As can be seen, the decrease of the removal rate in the presence of scavengers presents the following trend: benzoquinone > tert-butanol > ammonium oxalate, which is very similar to the results of Huang et al. [15]. Hence, the superoxide radical is the main reactive species during the photocatalytic degradation of MO, CR, and DB38.

**Fig. 12:**
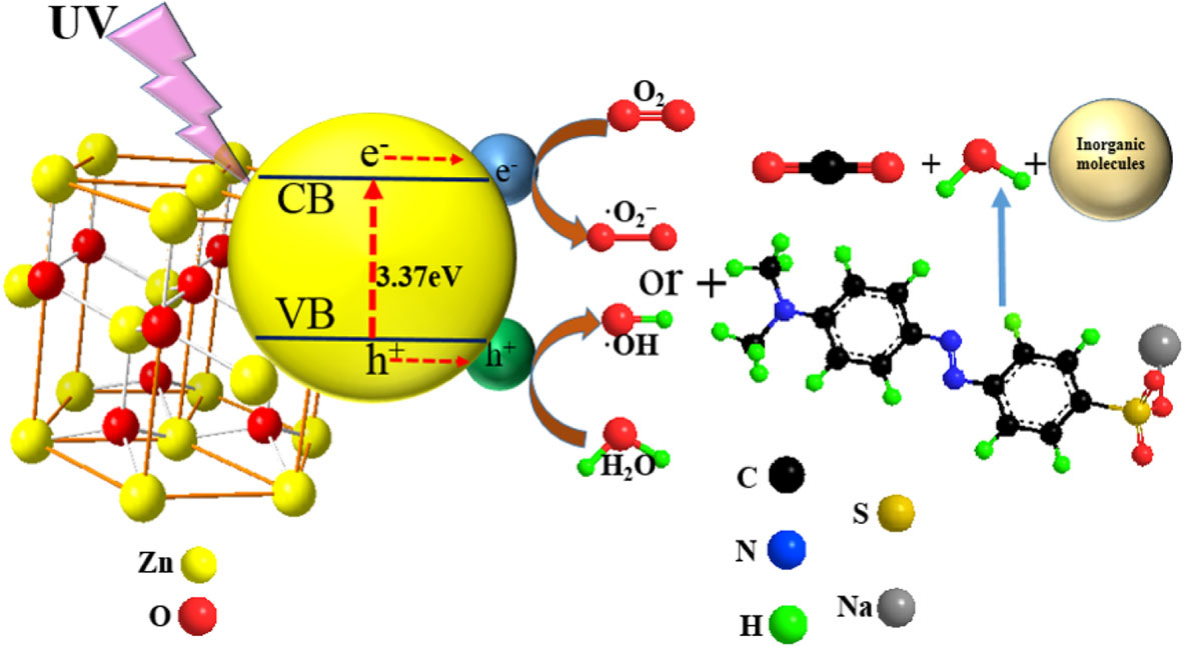
Mechanism for photocatalytic degradation of azo dyes on the ZnO photocatalyst under the UV irradiation

A mechanism for photocatalytic degradation of azo dyes on the ZnO photocatalyst under the UV irradiation is shown in Fig. 12. In a typical process, the electrons in the valence band transfer to the conduction band under UV irradiation of the photocatalyst. The corresponding energy is higher than the band gap of ZnO (3.37 eV), thereby promoting the generation of conduction band electrons (e^−^) and valance band holes (h^+^). The photo-generated holes could either directly oxidize adsorbed azo dyes or react with hydroxyl (OH^−^) or H_2_O to generate hydroxyl radicals (·OH). The photoelectrons reduce oxygen (O_2_) adsorbed on the photocatalyst surface into superoxide radical (·O_2_^−^). Finally, azo dyes were decomposed by the generated ·OH and ·O_2_^−^ [36]. The relevant reaction formulas are shown as follows:

$$ \mathrm{Z}\mathrm{n}\mathrm{O} + h v\to {\mathrm{h}}^{+} + {\mathrm{e}}^{-} $$

$$ {\mathrm{h}}^{+}+{\mathrm{OH}}^{-}\mathrm{or}\kern0.5em {\mathrm{H}}_2\mathrm{O}\to \mathrm{O}\mathrm{H} $$

$$ {\mathrm{e}}^{-}+\kern0.5em {\mathrm{O}}_2\to {{\mathrm{O}}_2}^{-} $$

$$ \mathrm{O}{\mathrm{H}}^{-}/\cdotp {{\mathrm{O}}_2}^{-}+\mathrm{azo}\ \mathrm{dyes}\kern0.5em \to \mathrm{C}{\mathrm{O}}_2 + {\mathrm{H}}_2\mathrm{O} + \mathrm{inorganic}\ \mathrm{molecules} $$

Meanwhile, through the scavenging radicals, the main degradation pathway of ZnO is the decomposition of MO, CR, and DB38 by ·O_2_^−^, which indicates that the mechanism of ZnO for the degradation of MO, CR, and DB38 is the same.

## Conclusions

The photocatalyst ZnO prepared by sol–gel method exhibits simple operation, flexibility, and high photocatalytic efficiency. The photocatalyst ZnO prepared with the composite ratio of 4:1 and calcination temperature of 400 °C presents satisfactory photocatalytic properties under UV irradiation. Based on the XRD and SEM results, the ZnO contains hexagonal wurtzite and the size of ZnO was 20–50 nm. The removal rate of azo dyes increased, with increased dosage of the photocatalyst and decreased initial concentration of the azo dye. The acidic condition is more favorable for degradation than alkaline condition. The degradation of azo dyes on ZnO was fitted by the first-order kinetics. Moreover, cycle experiment and radical scavenging tests on the degradation indicated that ZnO still remains at high photocatalytic activity and stability for a long time and superoxide ions are the main reactive species indicating that the azo dyes have the same degradation mechanism.
